# Phenolic Constituents from *Platycodon grandiflorum* Root and Their Anti-Inflammatory Activity

**DOI:** 10.3390/molecules26154530

**Published:** 2021-07-27

**Authors:** Wei Li, Hye Jin Yang

**Affiliations:** Korean Medicine (KM) Application Center, Korea Institute of Oriental Medicine, Daegu 41062, Korea; hjyang@kiom.re.kr

**Keywords:** *Platycodon grandiflorum*, lignol, phenolic, lignan, anti-inflammatory

## Abstract

Six lignols (**1**–**6**), including two new compounds (+)-(7*R*,8*R*)-palmitoyl alatusol D (**1**) and (+)-(7*R*,8*R*)-linoleyl alatusol D (**2**), along with four phenolics (**7**–**10**), a neolignan (**11**), three alkyl aryl ether-type lignans (**12**–**14**), two furofuran-type lignans (**15**–**16**), three benzofuran-type lignans (**17**–**19**), a tetrahydrofuran-type lignan (**20**), and a dibenzylbutane-type lignan (**21**) were isolated from the ethyl acetate-soluble fraction of the methanol extract of *Platycodon grandiflorum* (Jacq.) A. DC. root. The chemical structures of the obtained compounds were elucidated via high-resolution mass spectrometry and nuclear magnetic resonance (NMR) spectroscopy analyses. The obtained spectroscopic data agreed well with literature. Among the isolated compounds, eighteen (**1**–**7** and **11**–**21**) were isolated from *P. grandiflorum* and the Campanulaceae family for the first time. This is the first report on lignol and lignan components of *P. grandiflorum*. The anti-inflammatory effects of the isolated compounds were examined in terms of their ability to inhibit the production of pro-inflammatory cytokines IL-6, IL-12 p40, and TNF-α in lipopolysaccharide-stimulated murine RAW264.7 macrophage cells. Nine compounds (**4**–**6**, **12**, and **15**–**19**) exhibited inhibitory effects on IL-12 p40 production, eleven compounds (**1**–**6**, **12**, **15**–**17**, and **19**) exhibited inhibitory activity on IL-6 production, and eleven compounds (**1**–**6** and **15**–**19**) exhibited inhibitory effects against TNF-α. These results warrant further investigation into the potential anti-inflammatory activity and general benefits of the phenolic constituents of *P. grandiflorum* root.

## 1. Introduction

*Platycodon grandiflorum* (Jacq.) A. DC. is a perennial herb belonging to the Campanulaceae family that is mainly found in Northeast Asia, including countries such as China, Korea, and Japan. The root of *P. grandiflorum* (called Doraji in Korea, Jiegeng or Lingdanghua in China, and Kikyo in Japan) has been used as a food item in East Asia for thousands of years. In addition, *P. grandiflorum* has been used in traditional oriental medicine for treating lung and respiratory diseases such as cough, cold, bronchitis, asthma, and sore throat [1,2].

Results from previously published studies indicate that *P. grandiflorum* can have a relieving effect on cough and asthma; it has also been shown to exhibit extensive pharmacological effects, including anti-tumor, antioxidation, anti-inflammatory, and antibacterial activities; furthermore, it has been observed to afford protection against hypoglycemia and liver disease [3,4,5,6,7,8]. In the past few decades, *P. grandiflorum* has been reported to contain various chemical constituents such as triterpenoid saponins, flavonoids, phenolic acids, polyacetylenes, phytosterols, and polysaccharides. Platycodins, an oleanane-type pentacyclic triterpenoid saponin, is abundant in roots of *P. grandiflorum*, and they are the major bioactive constituents of this plant [1,2,9]. In addition, according to previous studies, platycodins identified in more than 70 from *P. grandiflorum* have a greatly diverse structure, and a wide range of biological and pharmacological activities, such as anti-tussive, anti-inflammatory, anti-cancer, anti-obesity, anti-fibrosis, and immune system-enhancing effects, have been reported [10,11,12,13]. Of these, platycodin D (PD) and platycodin D_3_ (PD_3_), the major platycosides of *P. grandiflorum*, exhibit significant anti-inflammatory activity. Moreover, previous studies have demonstrated that PD and PD_3_ regulate the production of pro-inflammatory cytokines, nitric oxide (NO), and secretion of tumor necrosis factor-alpha (TNF-α) in lipopolysaccharide (LPS)-stimulated murine RAW 264.7 macrophage cells [14,15].

**Figure 1:**
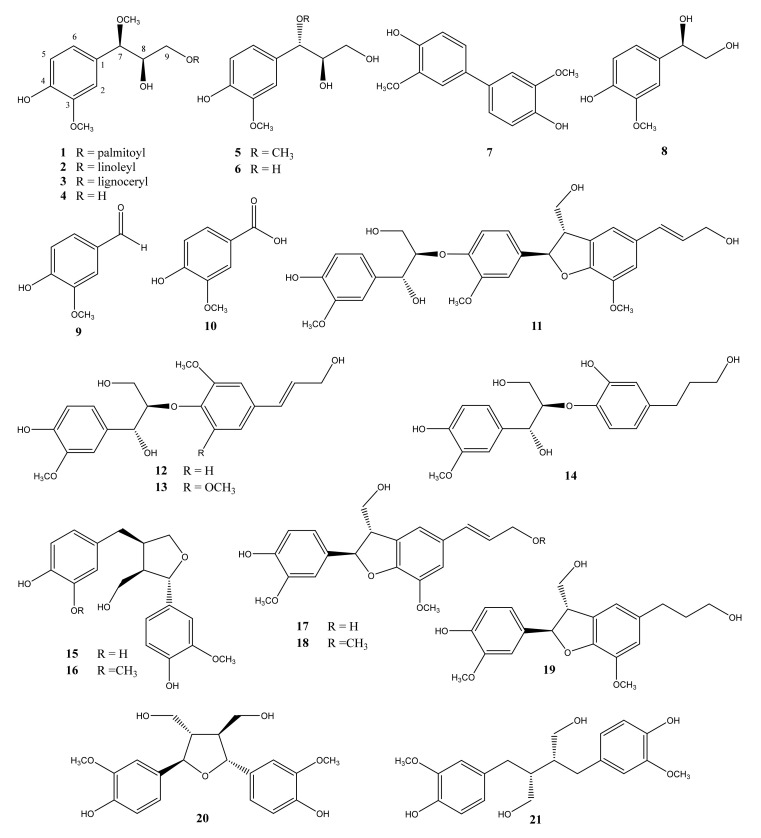
Structures of compounds **1**–**21** from the root of *P. grandiflorum*.

In practice, most pharmacological activity studies have been conducted on triterpenoid saponins, whereas research on the phenolic constituents of *P. grandiflorum* is lacking. Herein, we successfully isolated and elucidated the structures of 21 phenolic constituents present in methanol extracts of the *P. grandiflorum* root. In particular, the isolated compounds comprised two new and four known lignols, four phenols, a neolignan, nine lignans including two alkyl aryl ether type, two furofuran type, three benzofuran type, a tetrahydrofuran-type, and a dibenzylbutane-type (Figure 1). In addition, we report the results of experiments aimed at the inhibition of the pro-inflammatory cytokines IL-6, IL-12 p40, and TNF-α by the isolated phenol constituents of *P. grandiflorum* in lipopolysaccharide (LPS)-stimulated murine RAW264.7 macrophage cells.

## 2. Results and Discussion

### 2.1. Isolation and Structural Elucidation of Compounds ***1***–***21***

A series of twenty-one compounds, including six lignols (**1**–**6**), four phenolic compounds (**7**–**10**), a neolignan (**11**), three alkyl aryl ether-type lignans (**12**–**14**), two furofuran-type lignans (**15**–**16**), three benzofuran-type lignans (**17**–**19**), a tetrahydrofuran-type lignans (**20**), and a dibenzylbutane-type lignan (**21**), were isolated from the ethyl acetate-soluble fraction of the methanol extract of the root of *P. grandiflorum*. Their structures were identified as (+)-(7*R*,8*R*)-palmitoyl alatusol D (**1**), (+)-(7*R*,8*R*)-linoleyl alatusol D (**2**), (+)-(7*R*,8*R*)-lignoceryl alatusol D (**3**) [16], (+)-(7*R*,8*R*)-alatusol D (**4**) [17], (−)-(7*S*,8*R*)-alatusol D (**5**) [18], (+)-(7*S*,8*R*)-guaiacylglycerol (**6**) [19], 3,3′-dimethoxy [1,1′-biphenyl]-4,4′-diol (**7**) [20], (+)-4-hydroxy-3-methoxyphenylglycol (**8**) [21], vanillin (**9**), vanillic acid (**10**), 2-[4-[2,3-dihydro-3-(hydroxymethyl)-5-(3-hydroxy-1-propen-1-yl)-7-methoxy-2-benzofuranyl]-2-methoxyphenoxy]-1-(4-hydroxy-3-methoxyphenyl)-1,3-propanediol (**11**) [22], *threo*-(7*R*,8*R*)-1-(4-hydroxy-3-methoxyphenyl)-2-[4-[(*E*)-3-hydroxy-1-propenyl]-2-methoxyphenoxy]-1,3-propanediol (**12**) [23], wikstroemol (**13**) [24], threo-4,7,9,9′-tetrahydroxy-3,3′-dimethoxy-8-*O*-4′-neolignan (**14**) [25], (+)-lariciresinol (**15**) [26], 3′-demethyl-(+)-lariciresinol (**16**) [27], (−)-dehydrodiconiferyl alcohol (**17**) [28], hawthornnin G (**18**) [29], dihydrodehydrodiconiferyl alcohol (**19**) [30], (+)-neoolivil (**20**) [31], and (−)-secoisolariciresinol (**21**) [32], based on the consistency of their analytical data with those from available literature reports (Figure 1). Notably, compounds **1** and **2** are new compounds; in addition, eighteen compounds (**1**–**7** and **11**–**21**) were isolated from *P. grandiflorum* and from the Campanulaceae family in general for the first time.

Focusing on the two new compounds, compound **1** was isolated as a white amorphous powder. Its molecular formula was determined based on the presence of a pseudomolecular ion peak at an *m*/*z* value of 489.3190 [M + Na]^+^ (calcd. 489.3187) in the high-resolution electrospray ionization time-of-flight mass (HR-ESI-TOF-MS) spectrum. The proton nuclear magnetic resonance (^1^H-NMR) spectrum of compound **1** (Table 1) was characterized using three aromatic signals due to an ABX system [δ_H_ 6.70 (dd, *J* = 8.0, 1.9 Hz, H-6), 6.72 (d, *J* = 1.9 Hz, H-2), and 6.82 (d, *J* = 8.0 Hz, H-5)], two resonance signals due to oxymethine moieties [δ_H_ 3.80 (ddd, *J* = 6.1, 5.8, 3.4 Hz, H-8) and 3.97 (d, *J* = 6.1 Hz, H-7)], two resonance signals due to an oxymethylene group [δ_H_ 3.77 (dd, *J* = 11.0, 3.4 Hz, H-9a) and 3.99 (dd, *J* = 11.0, 5.7 Hz, H-9b)], and two resonance signals due to two different methoxy groups [δ_H_ 3.18 (s, 7-OMe) and 3.82 (s, 3-OMe)] that belong to a lignol moiety, which is the same as that present in compound **4**. The other resonance signals [δ_H_ 0.81 (t, *J* = 7.5 Hz, H-16′), 1.54 (m, H-3′), 1.17–1.30 (m, H-4′-15′), and 2.24 (t, *J* = 7.5 Hz, H-2′)] suggested the presence of a fatty acid moiety. Correspondingly, the ^13^C-NMR spectrum (Table 1) contained six aromatic signals corresponding to an ABX system of the aromatic rings [δ_C_ 109.1 (C-2), 114.4 (C-5), 120.9 (C-6), 129.2 (C-1), 145.9 (C-4), and 146.9 (C-3)], three lignol carbon signals [δ_C_ 64.5 (C-9), 73.6 (C-8), and 84.2 (C-7)], and a signal due to a carboxyl group [δ_C_ 173.7 (C-1′)], which proved that compound **1** is a lignol fatty acid ester [17]. The key heteronuclear multiple-bond correlation spectroscopy (HMBC) correlations between H-9 (δ_H_ 3.77 and 3.99)/C-1′ (δ_C_ 173.7), 7-OMe (δ_H_ 3.18)/C-7 (δ_C_ 84.2), and 3-OMe (δ_H_ 3.82)/C-3 (δ_C_ 146.9) are indicative of the connectivity between the lignol and fatty acid moieties, as well as the location of the methoxy groups.

**Figure 2:**
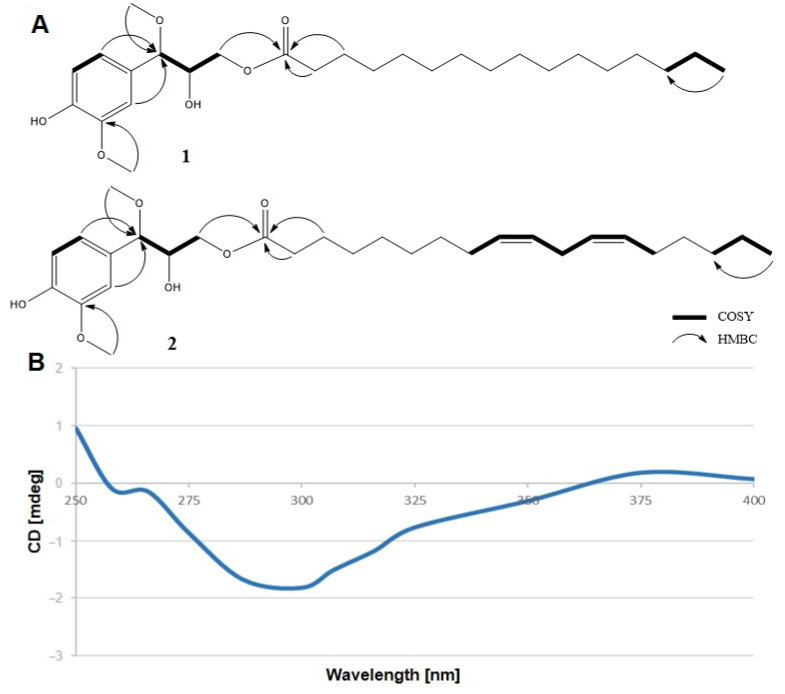
Key COSY and HMBC correlations of **1** and **2** (**A**) and the ICD spectrum of **1a** (**B**).

To determine the absolute configuration of compound **1**, the alkaline hydrolysis of **1** was conducted with 0.5% NaOH; consequently, palmitic acid and compound **1a** were produced. Notably, **1a** had the same structure as compound **4** did ((+)-(7*R*,8*R*)-alatusol D). The absolute configuration at C-8 of **1a** was determined by implementing Snatzke’s method [17]. The ICD spectrum of compound **1a** was characterized by a negative band at 300 nm (−1.8) (Figure 2B) [32], which is indicative of an 8*R* configuration at C-8. Based on the value of the coupling constant between H7 and H8 (*J* = 6.1 Hz), the absolute configuration at C-7 in compound **1** is 7*R* [17]. Based on these data, compound **1** was identified as (+)-(7*R*,8*R*)-palmitoyl alatusol D.

Compound **2** was isolated as a white amorphous powder. The molecular formula was established to be C_29_H_46_O_6_ based on the HR-ESI-TOF-MS peak observed at an *m*/*z* value of 513.3192 [M + Na]^+^ (calcd. 513.3187). The ^1^H-NMR and ^13^C-NMR spectra of this compound indicated that the lignol moiety in **2** was the same as that in **1**. The difference between compounds **2** and **1** contained the fatty acid moiety. The ^1^H-NMR spectrum of **2** (Table 1) comprised four overlapped olefinic proton signals at δ_H_ 5.27 (H-9′, 10′, 12′, and 13′). Correspondingly, the ^13^C-NMR spectrum of compound **2** (Table 1) comprised the signals due to four olefinic carbons at δ_C_ 129.7 and 130.2 (C-9′, 10′, 12′, and 13′). NMR evidence and GC-MS data thus suggest that **2** includes a linoleic acid moiety. Analogous to the case of compound **1**, the linoleic acid residue was attached to C-9, as indicated by the analysis results of the ^1^H–^1^H COSY and HMBC spectra (Figure 2). Therefore, compound **2** was determined to be (+)-(7*R*,8*R*)-linoleyl alatusol D.

### 2.2. Bioassay

Among the compounds present in the ethyl acetate-soluble fraction of the methanol extract of the root of *P. grandiflorum*, we identified those that exhibited anti-inflammatory activity by depressing the production of IL-12 p40, IL-6, and TNF-α in LPS-stimulated RAW264.7 cells. Preliminarily, the effect of compounds **1**–**21** (at a concentration of 100 μM) on the viability of RAW264.7 cells was evaluated by implementing the colorimetric MTT assay (Sigma, St. Louis, MO, USA.). According to the results of this assay, the tested compounds exhibited no cytotoxicity at the mentioned concentration (data not shown). Then, the effects of compounds **1**–**21** at various concentrations (1, 5, 25, 50, and 100 μM) on the production of IL-12 p40, IL-6, and TNF-α in LPS-stimulated RAW264.7 cells were investigated. Eight compounds (**4**–**6** and **15**–**19**) were observed to inhibit IL-12 p40, IL-6, and TNF-α production, with IC_50_ values determined to range from 5.0 to 60.6 μM; three compounds (**1**–**3**) were observed to inhibit the production of IL-6 and TNF-α, with IC_50_ values ranging from 6.5 to 20.2 μM; and compound **12** exhibited a weak inhibition of the production of IL-12 p40a and IL-6, with IC_50_ values of 56.2 and 62.7 μM, respectively (Table 2). The other nine compounds (**7**–**11**, **13**, **14**, **20**, and **21**) did not display any effect on the production of the mentioned cytokines at the indicated concentrations (IC_50_ > 100 μM). In these experiments, SB203580, a known inhibitor of p38 kinase, was used as a positive control; this compound inhibited IL-12 p40, IL-6, and TNF-α production with IC_50_ values of 5.3, 3.2, and 8.1 μM, respectively. Upon examination of the structure–activity relationship of the isolated compounds, we found that lignols (**4**–**6**) exhibited strong inhibitory activity on the three pro-inflammatory cytokines evaluated, whereas lignol fatty acid esters (**1**–**3**) only inhibited the production of IL-6 and TNF-α. This evidence can be preliminarily interpreted as indicating that the hydroxyl group on C-9 has a closely related effect on IL-12 p40; however, this hypothesis requires further research. A previous study revealed that alatusol E inhibited NO production in LPS-activated BV-2 cells, proving that lignol has a certain anti-inflammatory potential [33]. In contrast, among the 11 lignans (**11**–**21**), only furofuran-type (**15**–**16**) and benzofuran-type (**17**–**19**) lignans weakly inhibited the three pro-inflammatory cytokines considered. Previous reports have shown that various furofuran-type lignans significantly inhibit NO production [34]. However, there are no systematic reports on other inflammatory factors of furofuran-type lignans. Benzofuran-type lignans showed a strong inhibition of PGE_2_ synthesis [35], and some of them inhibited the pro-inflammation cytokines [36]. The present results also prove that benzofuran-type lignans can participate in the treatment of inflammation. These results not only reveal the anti-inflammatory activity of lignans in *P. grandiflorum* but also provide a side basis for research on the anti-inflammatory activity of lignans.

## 3. Materials and Methods

### 3.1. General Information

A Jasco DIP-370 automatic polarimeter was used to determine the optical rotation. The UV spectra were measured on a UNICO UV-2102PCS spectrophotometer (Unico, Dayton, NJ, USA). The NMR spectra were determined using a Bruker AM-600 spectrometer (Bruck Biospin, Fallanden, Switzerland). The LCQ advantage trap mass spectrometer (Thermo Finnigan, San Jose, CA, USA) was equipped with an electrospray ionization (ESI) source, and high-resolution electrospray ionization mass spectra (HR-ESI-MS) were obtained using an Agilent 6530 Accurate-Mass Q-TOF LC/MS system. Column chromatography was performed using silica gel (Kieselgel 60, 70–230, and 230–400 mesh, Merck, Darmstadt, Germany) and YMC RP-18 resins, and thin layer chromatography (TLC) was performed using pre-coated silica-gel 60 F_254_ and RP-18 F_254_S plates (both 0.25 mm, Merck, Darmstadt, Germany). GC-MS data were obtained with an Clarus 600 GC equipped with a 600T mass selective detector and a 30 m (0.25 mm i.d., 0.25 μm film) HP-5 ms capillary column (Agilent, Wilmington, Germany). All isolation solvents were purchased from Daejung (Si Heung, Korea).

### 3.2. Plant Material

Dried roots of *P. grandiflorum* were purchased in the Yangyeongsi herbal market, Daegu, Korea, in January 2016. The sample was botanically identified by the author (W., Li). A voucher specimen (AA-031) was deposited at the Korean Medicine (KM) Application Center, Korea Institute of Oriental Medicine, Daegu, Korea.

### 3.3. Extraction and Isolation

Dried roots of *P. grandiflorum* (2.0 kg) were extracted with methanol (MeOH) (10 L × 3) under reflux. The MeOH extract (300.0 g) was suspended in water and partitioned with *n*-hexane and ethyl acetate (EtOAc). The EtOAc fraction (21.0 g) was subjected to silica gel (4.0 × 30 cm) column chromatography with hexane:EtOAc, 10:1–5:1; hexane:EtOAc:MeOH, 1.5:1:0.15; chloroform (CHCl_3_):acetone:MeOH, 2:1:0.1; CHCl_3_-MeOH-H_2_O, 4:1:0.1; and MeOH, 100% to give 8 fractions (Fr. 1–8). The fraction 2 (3.2 g) was subjected to silica gel (1.5 × 80 cm) column chromatography with hexane-acetone (acetone 1–20%) elution solvent to give 9 sub-fractions. The fraction 2C was subjected to YMC (1 × 80 cm) column chromatography with MeOH-H_2_O (MeOH 70–95%) elution solvent to give compounds **1** (11.2 mg), **2** (9.6 mg), and **3** (11.8 mg). The fraction 2F was subjected to YMC (1 × 80 cm) column chromatography with MeOH-H_2_O (MeOH 60–95%) elution solvent to give compounds **4** (22.7 mg), **5** (7.4 mg), and **6** (7.0 mg). The fraction 2G was subjected to YMC (1 × 80 cm) column chromatography with acetone-H_2_O (acetone 60–90%) elution solvent to give compounds **8** (3.5 mg), **9** (70.6 mg), and **10** (28.7 mg). The fraction 4 (4.0 g) was subjected to silica gel (2.0 × 80 cm) column chromatography with CHCl_3_-acetone (acetone 6–35%) elution solvent to give 18 sub-fractions. The fraction 4A was subjected to YMC (1 × 80 cm) column chromatography with acetone-H_2_O (acetone 25–70%) elution solvent to give compounds **7** (7.8 mg) and **21** (14.2 mg). The fraction 4C was subjected to YMC (1 × 80 cm) column chromatography with acetone-H_2_O (acetone 20–70%) elution solvent to give compounds **12** (7.7 mg), **13** (11.6 mg), and **14** (7.8 mg). The fraction 4D was subjected to YMC (1 × 80 cm) column chromatography with MeOH-H_2_O (MeOH 30–80%) elution solvent to give compounds **15** (5.1 mg), **16** (20.7 mg), **17** (3.2 mg), **18** (20.6 mg), and **19** (8.4 mg). The fraction 4L was subjected to YMC (1 × 80 cm) column chromatography with MeOH-H_2_O (MeOH 10–70%) elution solvent to give compounds **11** (4.7 mg), **20** (8.2 mg), and **21** (22.2 mg).

*(+)-(7R,8R)-palmitoyl alatusol D (***1***)*: white amorphous powder; ${\mathrm{[\alpha ]}}_{D}^{25}$: –65.8 (*c* 0.05, MeOH); ^1^H NMR (methanol-*d*_4_, 600 MHz) and ^13^C NMR data (methanol-*d*_4_, 150 MHz), see Table 1; HR-ESI-MS: *m/z* 489.3190 [M+Na]^+^ (calcd. for 489.3187).

*(+)-(7R,8R)-linoleyl alatusol D (***2***)*: white amorphous powder; ${\mathrm{[\alpha ]}}_{D}^{25}$: –59.6 (*c* 0.05, MeOH); ^1^H NMR (methanol-*d*_4_, 600 MHz) and ^13^C NMR data (methanol-*d*_4_, 150 MHz), see Table 1; HR-ESI-MS: *m/z* 513.3192 [M+Na]^+^ (calcd. for 513.3187).

### 3.4. Alkaline Hydrolysis 

Compounds **1** and **2** (2.0 mg each) were treated with NaOH 1 N (2 mL) at 60 °C for 40 min, before the mixtures thus obtained were neutralized with HCl 1 N and extracted with CHCl_3_. The CHCl_3_ layers were then analyzed via silica gel thin-layer chromatography (TLC) using hexane–ethyl acetate (5:1 *v*/*v*) as the mobile phase; spots were visualized by spraying the TLC plate with 90% ethanol–H_2_SO_4_ (9:1, *v*/*v*) and then heating it at 180 °C for 2 min. The fatty acid moieties of compounds **1** and **2**, including palmitic acid (compound **1**) and linoleic acid (compound **2**), were analyzed via GC-MS, and palmitic acid (*t*R = 27.87 min) and linoleic acid (*t*R = 31.06 min) were confirmed through comparison with authentic samples.

### 3.5. Absolute Configuration

A 0.6–0.7-mg/mL stock solution of commercial Mo_2_(AcO)_4_ in commercial dimethyl sulfoxide (DMSO) was used. One part of the solution was evenly added into pure DMSO, and its circular dichroism spectrum was measured to be used as the baseline; the other part was added into DMSO alongside Mo_2_(AcO)_4_, and the molecular ratio of ligand to metal was about 1.0. After 30 min, the ICD spectrum of this solution was recorded [24]. Three absorption bands could be noticed in the ICD spectrum of Mo_2_(AcO)_4_ in the DMSO solution, the most obvious of which was observed at 305 nm, which, based on literature data, was attributed to a metal-to-ligand charge-transfer transition.

### 3.6. Cell Culture

The RAW 264.7 cells were obtained from the Korean Cell Line Bank (KCLB, Chongno-gu, Seoul, Korea) and maintained at 37 °C in RPMI 1640 medium supplemented with 10% fetal bovine serum (FBS) and 100 U/mL of penicillin in a humidified incubator containing 5% CO_2_. The cells were cultured in RPMI 1640 medium containing 10% FBS to 2 × 10^5^ cells/mL in 96-well tissue culture plates for 18 h; subsequently, they were pretreated with 0.4, 2, and 10 μM of the compounds to be tested 1 h prior to undergoing stimulation with LPS (1 μg/mL) for 24 h in an incubator.

### 3.7. Cell Viability

Cell viability was evaluated via the MTT method. Briefly, MTT was added to the cell culture medium. The supernatant was then removed, and the formazan crystals were dissolved in DMSO. The absorbance was measured at 570 nm. The percentage of dead cells was determined relative to the control group.

### 3.8. Measurement of Proinflammatory Cytokine Production

RAW 264.7 cells were incubated in 48-well plates containing 1 × 10^5^ cells per well; they were then treated with the isolated compounds **1**–**21** at the indicated concentration for 1 h before being subjected to stimulation with 10 ng/mL of LPS from *Salmonella minnesota* (Alexis, Famingdale, NY, USA). Supernatants were harvested 18 h after stimulation. The concentrations of murine TNF-α, IL-6, and IL-12 p40 in the culture supernatants were determined using ELISA (BD PharMingen, San Jose, CA, USA) according to the manufacturer’s instructions. The data were presented as means ± standard deviation of at least three independent experiments performed in triplicate.

### 3.9. Statistical Analysis

All data represent means ± SD of at least three independent experiments performed in triplicate. Statistical significance is indicated as determined via one-way ANOVA, followed by Dunnett’s multiple comparison test (*p* < 0.05) utilizing GraphPad Prism 6.0 (GraphPad Software Inc., San Diego, CA, USA).

## 4. Conclusions

The results of the present study afforded a comprehensive chemical assessment of the phenolic constituents of *P. grandiflorum* root. Notably, (+)-(7*R*,8*R*)-palmitoyl alatusol D (**1**) and (+)-(7*R*,8*R*)-linoleyl alatusol D (**2**) are newly discovered compounds, and eighteen other compounds were isolated from *P. grandiflorum* and the Campanulaceae family for the first time. To the best of our knowledge, this is the first comprehensive report on the phenolic components of *P. grandiflorum* and their anti-inflammatory activity. Results from this study may provide a scientific basis for the complement of anti-inflammatory components in *P. grandiflorum*.

## Figures and Tables

**Table 1:** ^1^H and ^13^C NMR spectroscopic data for compounds **1** and **2** in chloroform-*d*.

	1		2	
Position	δ_H_^a^ (*J*/Hz)	δ_C_^b^	δ_H_^a^ (*J*/Hz)	δ_C_^b^
1		129.2		129.2
2	6.72 d (1.9)	109.1	6.72 d (1.9)	109.1
3		146.9		146.9
4		145.9		145.9
5	6.82 d (8.0)	114.4	6.84 d (8.0)	114.4
6	6.70 dd (8.0, 1.9)	120.9	6.70 dd (8.0, 1.9)	120.9
7	3.97 d (6.1)	84.2	3.98 d (6.1)	84.2
8	3.80 ddd (6.1, 5.8, 3.4)	73.6	3.80 ddd (6.1, 5.8, 3.4)	73.6
9	3.99 dd (11.0, 3.4) 3.77 dd (11.0, 5.7)	64.5	4.00 dd (11.0, 3.4) 3.77 dd (11.0, 5.7)	64.5
1′		173.7		173.7
2′	2.24 t (7.5)	34.1	2.25 t (7.5)	34.1
3′	1.54 m ^c^	24.9	1.53 m	24.9
4′	1.17–1.30 m	29.1–29.7	1.18–1.27 m	29.1–29.8
5′	1.17–1.30 m	29.1–29.7	1.18–1.27 m	29.1–29.8
6′	1.17–1.30 m	29.1–29.7	1.18–1.27 m	29.1–29.8
7′	1.17–1.30 m	29.1–29.7	1.18–1.27 m	29.1–29.8
8′	1.17–1.30 m	29.1–29.7	1.94 m	27.2
9′	1.17–1.30 m	29.1–29.7	5.27 m	129.7
10′	1.17–1.30 m	29.1–29.7	5.27 m	129.7
11′	1.17–1.30 m	29.1–29.7	1.52 m	24.9
12′	1.17–1.30 m	29.1–29.7	5.27 m	130.2
13′	1.17–1.30 m	29.1–29.7	5.27 m	130.2
14′	1.17–1.30 m	31.9	1.95 m	27.2
15′	1.17–1.30 m	22.7	1.18–1.27 m	29.6
16′	0.81 t (7.5)	14.1	1.18–1.27 m	31.9
17′			1.18–1.27 m	22.6
18′			0.81 t (7.5)	14.1
3-OMe	3.82 s	56.0	3.84 s	56.0
7-OMe	3.18 s	56.7	3.19 s	56.7

Assignments were achieved by analyzing the HMQC and HMBC experiments; *J* values (Hz) are given in parentheses. ^a^ 600 MHz. ^b^ 150 MHz. ^c^ Overlapped.

**Table 2:** Anti-inflammatory effects of compounds **1**–**21** isolated from *P. grandiflorum* root on LPS-stimulated RAW264.7 cells.

	IC_50_ (µM) ^a^				IC_50_ (µM) ^a^		
	IL-12 p40	IL-6	TNF-α		IL-12 p40	IL-6	TNF-α
**1**	>100	8.1 ± 0.2	19.6 ± 0.5	**12**	56.2 ± 1.3	62.7 ± 3.1	>100
**2**	>100	6.5 ± 0.8	17.8 ± 1.1	**13**	>100	>100	>100
**3**	>100	9.2 ± 1.1	20.2 ± 0.9	**14**	>100	>100	>100
**4**	29.7 ± 3.2	5.0 ± 0.1	16.5 ± 3.0	**15**	29.7 ± 1.5	42.6 ± 0.7	20.6 ± 0.2
**5**	35.2 ± 0.7	5.1 ± 0.2	17.1 ± 2.1	**16**	21.3 ± 0.2	19.8 ± 0.8	18.9 ± 1.0
**6**	40.0 ± 0.1	7.7 ± 0.1	14.2 ± 0.2	**17**	10.1 ± 0.6	19.2 ± 1.8	18.1 ± 0.4
**7**	>100	>100	>100	**18**	60.6 ± 2.1	58.7 ± 2.9	49.3 ± 0.3
**8**	>100	>100	>100	**19**	22.3 ± 0.9	30.7 ± 2.0	46.9 ± 1.3
**9**	>100	>100	>100	**20**	>100	>100	>100
**10**	>100	>100	>100	**21**	>100	>100	>100
**11**	>100	>100	>100	SB203580 ^b^	5.3 ± 0.1	3.2 ± 0.2	8.1 ± 0.1

^a^ IC_50_ values for selected compounds are presented in columns IL-12 p40, IL-6, and TNF-α. Compounds exhibiting IC_50_ values > 100 µM were considered to be inactive. ^b^ Positive control.

## Data Availability

No new data were created or analyzed in this study. Data sharing is not applicable to this article.

## Sample Availability

Samples of the compounds **1**–**21** are available from the authors.
